# Song Morphing by Humpback Whales: Cultural or Epiphenomenal?

**DOI:** 10.3389/fpsyg.2020.574403

**Published:** 2021-01-15

**Authors:** Eduardo Mercado

**Affiliations:** Neural and Cognitive Plasticity Laboratory, Department of Psychology, University at Buffalo, Buffalo, NY, United States

**Keywords:** acoustic communication, cetacean, mysticete, self-organization, vocal learning, epiphenomenon

## Abstract

Singing humpback whales (*Megaptera noavaengliae*) collectively and progressively change the sounds and patterns they produce within their songs throughout their lives. The dynamic modifications that humpback whales make to their songs are often cited as an impressive example of cultural transmission through vocal learning in a non-human. Some elements of song change challenge this interpretation, however, including: (1) singers often incrementally and progressively morph phrases within and across songs as time passes, with trajectories of change being comparable across multiple time scales; (2) acoustically isolated subpopulations singing similar songs morph the acoustic properties of songs in similar ways; and (3) complex sound patterns, including phrases, themes, and whole songs, recur across years and populations. These properties of song dynamics suggest that singing humpback whales may be modulating song features in response to local conditions and genetic predispositions rather than socially learning novel sound patterns by copying other singers. Experimental and observational tests of key predictions of these alternative hypotheses are critical to identifying how and why singing humpback whales constantly change their songs.

## Introduction

Discussions of animal culture, defined as “shared behavior or information within a community acquired through some form of social learning from conspecifics” (Garland and McGregor, 2020), often highlight the incredible vocal skills of humpback whales (Laland and Janik, 2006; Allen, 2019; Whiten, 2019). For instance, Rendell and Whitehead (2001a) identify humpback whale songs as one of the strongest examples of cetacean culture, a point echoed by Laland and Hoppitt (2003) and Janik (2014). The main reason humpback whales have garnered so much attention in the context of culture is because of the unique ways in which they change their songs throughout their lives. As Garland and colleagues note, “the level and rate of change is unparalleled in any other nonhuman animal and thus involves culturally driven change at a vast scale” (Garland et al., 2011, p. 690). Payne (2000) compared the process of whale song change to linguistic drift, but much faster, noting that within 1 decade songs produced by a population can change so much that it is not possible to recognize how they relate to earlier versions.

The songs of humpback whales are often described as being highly sophisticated communicative displays, possessing a multilayered hierarchical structure (Payne and Payne, 1985; Rekdahl et al., 2018). Song features show regional specificity, such that in a given year, different populations can be distinguished based on the songs being produced (Winn et al., 1981). Individual singers gradually change song properties throughout their lives, within and across years, never settling on a stable, favored song (Guinee et al., 1983; Payne et al., 1983; Payne and Payne, 1985). Notably, singers in a particular area change their songs in parallel, leading to the inference that singers are copying each other’s songs. The driving force behind such copying is believed to be an acoustic competition that reveals a singer’s reproductive fitness to other whales (Payne, 2000; Herman, 2017). In summary, the current consensus view about singing humpback whales is that all singers continuously and irreversibly, modify their song content throughout their adult lives, either by introducing new song elements or by copying new elements heard from other whales, so that they can maximize mating opportunities. In contrast, I hypothesize that mechanisms other than cultural transmission are the primary drivers of song transformations, and more generally that variations in song across years are epiphenomenal (i.e., an incidental byproduct of song production and reception mechanisms) rather than cultural.

Numerous proposals have been made for why and how humpback whales change their songs over time (for review, see Parsons et al., 2008), all of which start with the assumption that cultural transmission plays a key role. A few researchers have questioned this assumption, however. For example, demonstrations of cultural transmission of vocal behavior by birds depend on showing that vocal traditions are characteristic of groups, socially learned, and fairly stable across generations (Freeberg, 2000). Freeberg (2001) noted that none of these processes have been definitively shown to occur in singing humpback whales, making evidence relating song changes to cultural processes difficult to interpret. Specifically, he points out that “just because we can measure differences does not mean the animals perceive or care about those differences (p. 334).”

The proposal that humpback whales socially learn their songs through processes of cultural transmission is a hypothesis, hereafter, referred to as the *song-copying hypothesis*. The song-copying hypothesis is closely related to a second hypothesis – that humpback whale songs function as a sexual advertisement display (Payne and McVay, 1971). According to the sexual advertisement hypothesis, the reason why male humpback whales copy songs is because better songs yield more and/or higher quality offspring, where better means preferred by females, envied by other males, or both. In combination, these two hypotheses attempt to explain both why humpback whales sing structurally complex songs and why they continuously modify their songs over time. Neither hypothesis requires that songs change over time, since there are other cetaceans like dolphins that copy sounds without constantly changing them and without using sounds as sexual advertisement displays (Mercado et al., 2014), and there are other mammals such as red deer that use sounds as vocal reproductive displays without copying sounds or changing them over time (Reby et al., 2005). In fact, no other mammals use constantly changing sound sequences as a sexual display (with the possible exceptions of popular musicians and bowhead whales; Stafford et al., 2018), making humpback whales a biological anomaly, hypothetically. Neither hypothesis has ever been tested in any substantive way. The only evidence providing any support for these hypotheses are the phenomena that they were initially proposed to explain (see also Mercado, 2018). Here, it is argued that the song-copying hypothesis is neither necessary nor sufficient for explaining how humpback whales change their songs over time and that mechanisms other than cultural transmission can better account for known temporal variations in the acoustic characteristics of humpback whale songs.

## Morphing of Song Phrases

Fully assessing the song-copying hypothesis would require a variety of costly and time-consuming field experiments. It is possible to partially evaluate the viability of this hypothesis, however, through closer examination of the phenomena that originally led to it. Specifically, one can obtain clues to the mechanisms of song change by closely examining when and how songs change. Surprisingly, researchers have rarely attempted to do this, instead opting to compare either representative spectrograms of sound patterns or symbol sequences representing subjective impressions of spectrograms. For instance, Winn and Winn (1978) subjectively analyzed yearly and within-year changes in songs by comparing spectrographic images of representative sound sequences (“phrases”), and verbal descriptions of phrases, from 8 consecutive years (see also Cato, 1991). Payne and Payne (1985) compared songs across years by labeling repeated phrases (“themes”) with different graphical patterns, switching to new patterns when phrases were sufficiently dissimilar (subjectively judged). Later analyses of song change used more objective metrics (e.g., Cerchio et al., 2001; Eriksen et al., 2005; Garland et al., 2011), but still depended heavily on subjective impressions of phrase similarities and differences, typically denoted using alphanumeric labels (see also Cholewiak et al., 2013). Subjective symbolization of sound patterns is limited as an approach to characterizing how whales change songs because it discards many of the features that whales actually modify and replaces them with abstractions that reveal little about the acoustic features of the sequences being produced (Kershenbaum et al., 2016; Mercado and Perazio, in press). Attempts to quantitatively assess changes in whale songs over time revealed that some acoustic features of songs change more rapidly across years than others (Payne et al., 1983; Mercado et al., 2003, 2005; Green et al., 2011). Although past research studies have differed in their methodologies, one phenomenon that is consistently noted is that singers gradually change the acoustic features of the individual sounds (“units”) within phrases over time. Changes in the units within phrases are apparent both within songs and across years (Cato, 1991; Maeda et al., 2000; Arraut and Vielliard, 2004; Mercado et al., 2005).

**Figure 1:**
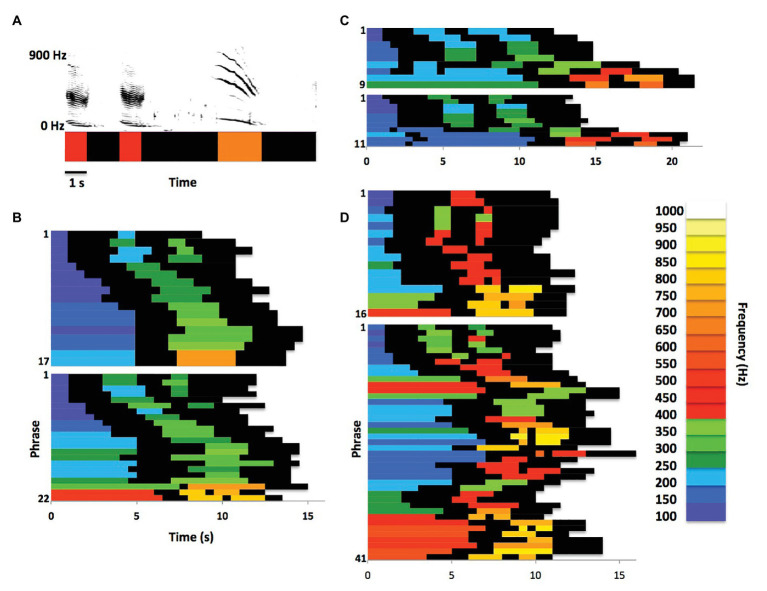
Phrase morphing within themes. **(A)** Traditional spectrographic depictions of humpback whale song phrases can also be represented as pitch vectors (Hyland Bruno and Tchernichovski, 2019), with colors representing peak frequencies of units to highlight spectral and temporal patterns. **(B)** Payne and McVay’s (1971) Theme 1 (recorded in Bermuda in 1964) in raster plot form shows progressive changes in unit duration, number, and frequency content across phrase “repetitions”; the two plots show Theme 1 from consecutive songs produced by one singer, illustrating intra-individual variations in theme and phrase production within a shifting theme (data from Payne and McVay, 1971, Figure 6, Whale I). **(C)** Raster plots from a second singer producing two consecutive versions of Theme 1 (recorded in Bermuda in 1963) illustrate inter-individual differences and/or cross-year differences in phrase duration, as well as in specific acoustic features of units (data from Payne and McVay, 1971, Figure 6, Whale II). Despite these differences, similarities in morphing trajectories and phrase structure across singers/years are apparent. **(D)** Plots from a third singer (recorded in Bermuda in 1963) show how variable two consecutive versions of Theme 1 can be, differing not only in the number of phrase repetitions, but also in the trajectory of phrase morphing and in the distribution of unit features and timing. The second raster plot also raises the question of how one might distinguish a longer duration Theme 1 from Theme 1 produced twice in a row (data from Payne and McVay, 1971, Figure 6, Whale III).

Payne and McVay (1971) were the first to report that phrases produced by singing humpback whales often systematically change as they are being repeated. Many of these changes qualify as natural sound morphing, in which one or more acoustic features gradually shift as a sound is repeated within a sequence (Caetano and Rodet, 2013). Figure 1 illustrates one way that singing humpback whales morph phrase elements while singing. Payne and Payne (1985) described sections of songs in which singers gradually morphed units across phrases as “shifting themes.” Specifically, they defined shifting themes to be “themes in which successive phrases evolve progressively from one form to another. As phrase follows phrase, units within them gradually shift in frequency and/or form, duration, or numbers, or are delivered at a slower or faster rate (p. 99).” Payne and Payne identified one or two shifting themes in recordings from every year they analyzed; suggesting that phrase morphing was a consistent feature of humpback whale songs. Singers morph both units and phrases along multiple acoustic dimensions, including duration, pitch, spacing, rhythm, frequency modulation, timbre, intensity, and number of units (Mercado and Perazio, in press). Recent detailed comparisons of morphing trajectories revealed that singers produce comparable within-song transformations across years and populations (Mercado and Perazio, in press). Payne and Payne described shifting themes as being one of three types produced by singers, the other two being “unorganized” and “static.” It is perhaps more accurate to describe both shifting and static themes as points along a continuum of phrase production ranging from substantial morphing (shifting) to very little morphing (static), because no phrase repeated by a singer is acoustically identical to its predecessor and subtle morphing is often evident at the beginning and end of even the most static themes (Mercado et al., 2003).

Early spectrographic images of shifting themes also revealed that the degree of phrase morphing varies within song sessions, and even across consecutive songs (Payne and McVay, 1971). Traditionally, researchers have described these differences as variations in the number of times that phrases within a particular shifting theme were repeated. Side-by-side comparisons of consecutive themes reveal, however, that when one version of a theme contains less phrase repetitions than a subsequent instance of that theme, then the rate of morphing varies across the two themes (e.g., see Figure 6 in Payne and McVay, 1971). In other words, morphing trajectories and rates vary with the number of repetitions within a theme as opposed to being an obligatory component of repetition. Consequently, the eighth phrase in a 16-phrase shifting theme is acoustically different from the eighth phrase in a subsequent 40-phrase version of that same theme (see Figure 1D), implying that: (1) singers morph phrases differently early on in a theme when initiating a longer series of repetitions; and (2) singers have some flexibility in terms of how they morph phrases within and across shifting themes.

**Figure 2:**
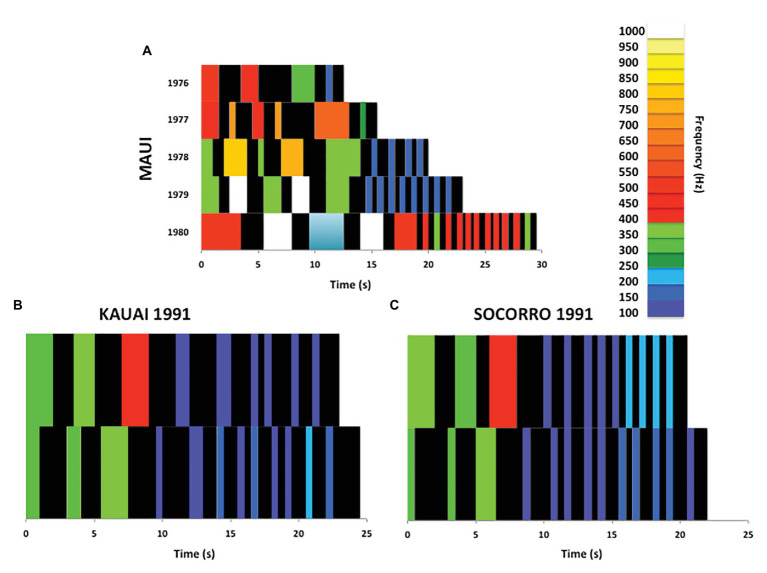
Phrase morphing within and across years. **(A)** A raster plot of Payne et al. (1983) Theme 5 (recorded off the coast of Maui in 5 consecutive years) shows progressive morphing of acoustic features, including expansion of unit durations, bifurcation of units (increasing their number), as well as shifts in the pitches produced within the phrase (data from Payne et al., 1983; Figure 5). The trajectories of acoustic transformations of this theme across years are comparable to those present within a shifting theme (compare with Figure 1). **(B)** Comparisons of pitch vectors depicting Cerchio et al.’s (2001) Theme 2a recorded off the coast of Kaui in either early February (top row) or early April (bottom row) reveal bifurcations of shorter duration units, reductions in the duration of a subset of longer duration units, and a shift to slightly different frequencies (data from Cerchio et al., 2001; Figure 3A). **(C)** Comparisons of this same theme recorded during the same time periods off of Isla Socorro (~4,800 km away) show highly similar phrase morphing trajectories, with inter-unit intervals changing in similar ways across the two regions (data from Cerchio et al., 2001; Figure 3A). Parallel phrase morphing across such long distances is inconsistent with either copying of innovators or with changes being introduced by copying errors. Note also that the within-season changes in phrase properties reported by Cerchio et al. (2001) are comparable to those reported by Payne et al. (1983) between 1978 and 1979, shown in **(A)**, suggesting that singers may transform phrases similarly across decades.

Payne et al. (1983) conducted quantitative analyses of phrase morphing by singing humpback whales in Hawaiian waters both within and across consecutive years. They focused their phrase analyses primarily on changes in the number, duration, and configuration of units within phrases. These analyses revealed gradual morphing of phrases over both months and years, with trajectories and rates of morphing varying across themes, months, and years – these variations in morphing were cited as evidence that the changes were cultural rather than environmental (see Figure 2A for examples of morphing across years). The progressive yearly changes in phrases that they reported were comparable to the kinds of changes that they observed within years, which were comparable to the acoustic transformations that occurred within shifting themes. Payne and colleagues noted that the changes in phrases were complex and asynchronous. Different phrases or components of phrases called “subphrases,” morphed in different ways and at different rates. Nevertheless, they described all of these changes as appearing “to follow set rules of progressive change.” Other more rapid changes in humpback whale songs that occur across years (referred to as “song revolutions,” see Noad et al., 2000; Allen et al., 2018) may also involve morphing of phrases (Garland et al., 2017; Allen et al., 2019). However, because revolutions were identified based on comparisons of symbolic transcriptions of songs rather than through direct acoustic comparisons of units, it is difficult to evaluate whether new phrases were morphs of earlier phrases.

**Figure 3:**
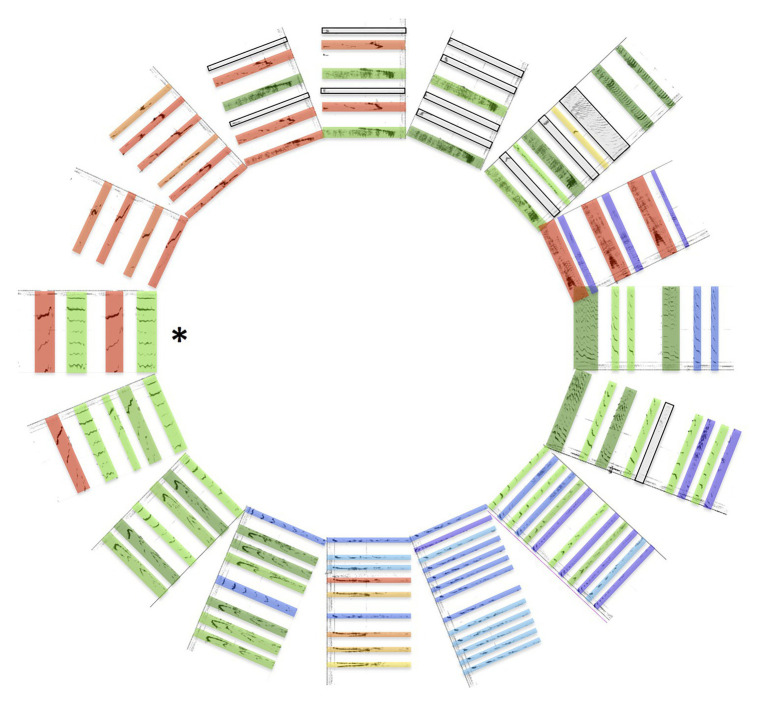
Phrase morphing within songs. Spectrograms of successive patterns within an archival recording of humpback whale song obtained from the Macaulay Library (catalog number 110858; recorded by Perkins in 1973, British Virgin Islands) arranged to highlight the cyclical nature of song production (colored bars indicate peak frequencies, as in Figures 1, 2), reveal cross-theme morphing of multiple acoustic features. The entire cycle was segmented by identifying a single distinctive pattern and then selecting each subsequent pattern such that it was aligned in duration and timing to the previous segment as closely as possible (i.e., no attempt was made to identify phrases). Sixteen segments were sampled from 64 total comprising a single cycle to illustrate changes over time within the cycle (see also Schneider and Mercado, 2019; Mercado and Perazio, in press). Note that alternating spectrotemporal patterns are prominent throughout the cycle and that adjacent patterns share multiple acoustic properties in common, even as the duration, number, and peak frequencies of units within patterns progressively shift throughout the cycle. The asterisk indicates a pattern that was repeated 10+ times with minimal modifications.

Traditionally, researchers analyzing humpback whale songs have classified sets of repeated “phrase types” that occur in a predictable order within songs as themes, with the initiation of a new theme signaled by a switch to a “new phrase type” (Cholewiak et al., 2013). What qualifies as a new phrase type is often subjective and may vary across investigators, such that different researchers analyzing identical (Mercado et al., 2003), or similar (Thompson and Friedl, 1982; McSweeney et al., 1989), recordings of songs may identify different numbers of themes within those songs. As an extreme example, Payne and McVay (1971; Figure 8, Whale III) classified a single unit as an instance of a theme. Phrase types derived from quantitative analyses of perceptually-based unit categories (e.g., Garland et al., 2012; Allen et al., 2017) are similarly problematic because different unit categories will generate different symbolic sequences. Segregating phrases in terms of themes facilitates analyses of ordered cycles within song sessions as well as across singers, and can simplify analyses of temporal variations in these cycles (Frumhoff, 1983). This approach makes it difficult, however, to detect progressive acoustic changes that are occurring within song cycles (Mercado and Handel, 2012; Perazio and Mercado, 2018). Consequently, few analyses have described examples of cross-theme morphing of units or unit sequences. Mercado et al. (2010), Mercado and Sturdy (2017), and Mercado and Perazio (in press) noted that spectral features of units appeared to be gradually shifting throughout entire songs. Automated analyses of unit sequences further revealed that some acoustic properties of units were relatively stable within a song cycle while others were progressively changing across themes (Mercado and Sturdy, 2017). This finding led to the discovery of “drone units,” acoustically similar units that recurred across multiple themes, often at regular intervals (Mercado, 2016). Collectively, these past findings suggest that singing humpback whales are not limited to morphing phrases within shifting themes, but may also do so throughout entire song cycles. For at least some humpback whale songs, the entire song cycle is arguably one long shifting theme (Mercado and Perazio, in press), with asynchronous morphing (or deletion) of phrases determining the order of pattern progression with the cycle (Figure 3).

One reason why morphing of phrases across themes has received relatively little attention in past analyses of humpback whale songs is because of the widespread use of the hierarchical framework that Payne and McVay (1971) proposed for describing structure within songs (Suzuki et al., 2006; Cholewiak et al., 2013). This approach treats themes within songs as discrete, independent events, like horses on a merry-go-round. From this perspective, acoustic similarities between themes are less relevant than any features that might make a theme subjectively distinctive from other themes. Song production can alternatively be viewed as a heterarchical process in which cycles of respiration and air recirculation interact in ways that constrain the form of repeating phrases (Mercado and Handel, 2012; Mercado and Perazio, in press). Sounds produced bidirectionally, during both the inspiration and expiration of air, typically show characteristic differences in acoustic features (e.g., the hee-hawing production of brays by donkeys). Alternating acoustic properties of units and unit clusters consistent with bidirectional sound production is evident within humpback whale song phrases (Cazau et al., 2013; Mercado and Perazio, in press). When songs are analyzed in ways that preserve heterarchical structure, clear evidence of “cross-theme” phrase morphing becomes evident (Figure 3). Singers maintain continuity in rhythmic structure (Schneider and Mercado, 2019), unit duration and number, pitch alternation, and pitch shifting as they progress through a song cycle. Although there are periods when singers are morphing patterns more rapidly (traditionally referred to as shifting themes) or more slowly (“static” themes), the waxing and waning of temporal and spectral features of patterns appears to follow smooth trajectories throughout a song cycle (Mercado and Perazio, in press). For example, in the song cycle shown in Figure 3, longer duration units gradually bifurcate into pairs of shorter duration units, which later merge back into individual longer duration units, only to split and merge again later in the cycle. Continuous shifts in the frequency content of units are also present, with the singer focusing more on lower- or higher-pitched units during different parts of the cycle (Perazio and Mercado, 2018; Mercado and Perazio, in press). If one compares phrases produced more than 5 min apart in a song cycle, the patterns are likely to be subjectively distinctive, and can thus be designated as different themes. Partitioning the song cycle in this way is arbitrary, however, and obscures the fact that singers are continuously morphing some features of consecutive phrases while preserving others.

In summary, analyses of phrase morphing across song cycles (i.e., progressive evolution of songs) over the past 50 years have revealed that: (1) singers gradually change acoustic elements of their sound sequences within and across themes as well as across weeks, months, and years; (2) phrases within songs can morph along multiple acoustic dimensions in parallel, and subjectively distinctive phrases can morph in different ways and at different rates; (3) individual singers can vary the rate at which they morph phrases within a song session, but whales within a population morph phrases collectively over time at a “group rate” such that singers in the population are generally producing similar sequences of phrases; and (4) the ways in which singers morph phrases are similar across multiple time scales, although the trajectories of phrase changes vary in rate and extent across years.

These observations provide the primary evidence of cultural transmission of song characteristics between singing humpback whales. This evidence, despite being correlational, is considered compelling by many researchers because the rapid changes in song structure in some years but not others, combined with the synchronized changes across individual singers, seems to rule out any possible genetic or environmental factors that could drive the changes (Payne et al., 1983; Rendell and Whitehead, 2001b; Laland and Janik, 2006). Essentially, the argument is that no other possible mechanism is left to account for the observed patterns of change in humpback whale songs other than cultural transmission of song characteristics through vocal imitation and innovation. This “method of exclusion” or ethnographic approach has been critiqued by Laland and Janik (2006) and Laland et al. (2009), because: (1) it is infeasible to rule out that some unknown genetic or ecological factors explain the variance attributed to culture and (2) genes, ecology, and learning always interact in ways that affect behavior. A simpler empirical argument against invoking cultural transmission to explain song transformations by humpback whales is that lone singers commonly morph phrases within shifting themes (i.e., within a song cycle) in ways that parallel progressive changes in themes across years. Given that phrase morphing across songs is acoustically similar to phrase morphing within songs, it stands to reason that similar production mechanisms could potentially account for both transformations. This account is more parsimonious than cultural explanations for song change because it attributes both transformational phenomena to a single mechanism that does not require social learning.

If singers are predisposed to morph phrases or units along predictable trajectories (both within and across songs), then this would naturally lead to converging transformations of song forms across individuals who progress from similar starting points. In this scenario, progressive changes to songs should be similar across populations, with similar patterns of morphing occurring in different locales and time periods. In contrast, the song-copying hypothesis predicts that song transformations should diverge across populations that are not in acoustic contact because of innovations and accumulating copying errors introduced by individual singers. The following section summarizes evidence for and against these two alternatives.

## Recurring Song Elements Within and Across Populations

Yearly changes in humpback whale song are often described as being progressive (or revolutionary) and irreversible (Winn and Winn, 1978; Payne et al., 1983; Payne and Payne, 1985; Eriksen et al., 2005; Garland et al., 2017). Songs recorded from different populations are also typically described as being “quite different in content” (Winn et al., 1981; Payne, 2000). Several researchers have proposed that observed differences in humpback whale songs over time and across populations arise through improvisation by particularly fit or creative singers (Payne, 2000; Cerchio et al., 2001; Noad et al., 2004). According to this interpretation, older themes are successively replaced by new themes to create novel songs that are unique to each population every year. Some themes may be replaced by similar themes or even persist unchanged for several years, but ultimately all themes will be discarded in favor of more fashionable/functional themes. And, once a theme is discarded, there is no reason why it should reappear because there should be no singers modeling production of it and the old theme would no longer qualify as an innovative addition.

Past assessments of the novelty of songs and themes across years have largely been based on subjective impressions of song recordings: both aural impressions of recordings and visual impressions of spectrographic representations. There is no way to know how humans’ impressions relate to the percepts of singing whales, and consequently no way to objectively identify what qualifies as novel or familiar to a humpback whale. Nevertheless, it seems probable that singers would be more likely to recognize a theme that is acoustically similar to other themes it has experienced in the past. There is clear evidence that a subset of acoustically similar themes persist across decades, even if they are not present in all years, and that themes that persist in one population are also likely to show up intermittently in other populations. For instance, both Winn and Winn (1978) and Payne and Payne (1985) identified the “surface ratchet” theme in songs spanning a decade. Although this theme was not present in all years analyzed, it was consistently structured and has subsequently been identified in every population of singing whales that has been analyzed for multiple years. Payne and McVay (1971) designated the surface ratchet theme as a shifting theme; Winn and Winn (1978) pointed out that in some years it included tonal units, but that in other years it did not. This theme has often been classified as the “first” or “last” theme in humpback whale songs because of its association with surfacing. It is thus well-established that at least one theme repeatedly reappears after being dropped, and does so in multiple populations worldwide.

**Figure 4:**
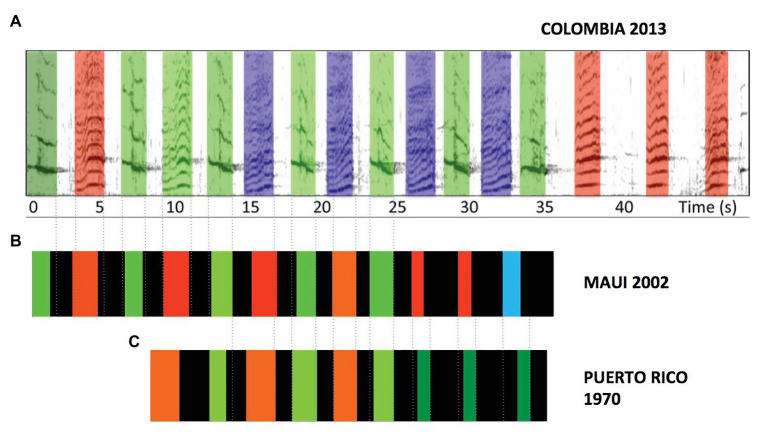
**(A)** A prevalent 16-unit phrase recorded in Colombia in 2013 includes repetitive alternation of frequency-modulated tonal units followed by a trio of acoustically similar units (data from Perazio et al., 2018). **(B)** Phrases with highly similar acoustic structure were recorded in Maui in 2002 (data from Mercado, 2016), and **(C)** in Puerto Rico in 1970 (data from Winn et al., 1970). Note that in addition to the similar alternation of units followed by a triplet of tonal units, phrases show highly similar modulation of unit durations and inter-unit intervals as the phrase progresses, such that unit production is temporally aligned across populations (dotted lines). The phrases differ mainly in terms of the number of alternations and in the peak frequencies of individual units.

The surface ratchet is not the only theme that has been dropped by singers only to reappear at later periods. Mercado et al. (2003) noted two others that have been reported in multiple populations. Figure 4 illustrates variants of phrases from one of these themes, a theme identified by both Winn et al. (1970) and Payne and McVay (1971) that has subsequently been reported in several other populations (e.g., Perazio et al., 2018). Much like the surface ratchet theme, this theme comes in two flavors depending on whether or not a subphrase of tonal units is included. Also like the surface ratchet theme, the units that make up this phrase are not acoustically identical either across populations or across years (Darling et al., 2019). Nevertheless, variants of this theme are structurally more similar than the beginning and ending phrases of shifting themes and it is unlikely that the specific combination of features that they share could have arisen through independent innovations. Finally, this theme often contains the largest number of phrase repetitions within songs where it appears (Payne and McVay, 1971; Mercado et al., 2003; Perazio et al., 2018; Darling et al., 2019).

Payne and McVay (1971) originally described the songs they analyzed as consisting of three main sections: (1) the surface ratchet theme, (2) clusters of rapidly produced, short-duration units, and (3) sustained units monotonously alternated (similar to the theme illustrated in Figure 4). All three of these “main sections” are recurring themes that have been intermittently identified in songs across populations and years (Mercado et al., 2003). Recent comparisons of phase morphing by singers across decades and populations revealed not only that singers show similar morphing trajectories over time and space, but also that entire songs may be replicated (including detailed spectrotemporal patterning within phrases) across distant populations and long time spans (Mercado and Perazio, in press). The fact that singers in acoustically isolated populations are consistently using recurrent themes produced in the same order and with similar phrase morphing trajectories argues strongly that a non-reversible process of accumulating, culturally transmitted modifications (or copying errors) is not what drives the progressive changes evident within humpback whale songs.

## Understanding the Nature of Humpback Whale Song Transformations

Payne and McVay (1971) and Payne et al. (1983) were the first to describe the acoustic properties of humpback whale songs as slowly evolving, and to suggest that the mechanism driving such changes was cultural. The main phenomena they noted as evidence of this interpretation were: (1) changes in songs are rapid, complex, continuous, and non-reversing, and so could not be driven by genetic changes; (2) the rate and types of changes observed varied across years, so were unlikely to be driven by seasonal factors; (3) songs produced at the beginning of a breeding season were highly similar to those produced at the end of the previous season, and the greatest changes appeared when singing was most prevalent, suggesting that changes were not the result of memory errors; and (4) the most pronounced changes appeared to be adopted by all singers in parallel, suggesting that singers were socially transmitting changes through acoustic contact. In short, how singers morphed song characteristics over time led to the inference that humpback whales must be learning what to sing by copying one another. Later work showed that song forms that were initially rare could rapidly become prevalent within a population (Noad et al., 2000), that singers sometimes adjust their song production upon hearing other songs (Cholewiak et al., 2018), and that singers may embellish existing phrases over time (Allen et al., 2018). These observations continue to be the main data cited as evidence of communicative culture and cultural transmission in humpback whales.

The first three phenomena noted above can potentially be explained as resulting from individual learning processes. But, this would not account for convergence across singers. Shared used of vocalizations alone could easily be explained as an inherited capacity. However, it is difficult to imagine how such continuous, complex changes in songs that occur synchronously in whales of various ages might be genetically encoded, given that each newly developed singer would need to sing a song that is the “same”^1^ as those being sung by whales with decades more singing experience. If singing humpback whales are learning songs by copying other singers, then this raises the question of when a singer will copy other singers it has heard, as well as the question of why songs change at all if singers are all copying what they hear other whales doing? Whale researchers have speculated that some songs are superior to others and that whales that hear songs that are “better” than the ones they are singing will attempt to copy those songs (Noad et al., 2000; Darling and Sousa-Lima, 2005; Garland et al., 2011). Because this process alone would ultimately result in all whales singing the same best song, it has further been suggested that songs that differ from the norm are better, leading to a kind of vocal arms race (Cerchio et al., 2001; Garland et al., 2017). However, in a runaway vocal competition scenario, one might expect to see innovative songs competing to become the new norm, which does not seem to occur. Consequently, yet another speculative assumption must be introduced. Namely, that not all innovations will make a song “better,” thus reviving the original question of when a singer will deem another singer’s song worthy of copying, combined with the question of what circumstances might lead an individual whale to attempt to be vocally creative. Currently, there is no way to objectively classify any song produced by a humpback whale in terms of its quality or innovativeness.

Current explanations for progressive changes in humpback whale songs attempt to apply principles of biological evolution (e.g., sexual selection) to the vocal actions of individual whales by proposing that only the “fittest” themes survive, and then only for a limited time (Parsons et al., 2008). It is unclear, however, whether processes of mate selection can explain the kinds of changes observed in the songs of humpback whales. First, this kind of process can only explain synchronization of song changes if there are “leaders” that all whales in a population are following. Otherwise, as noted above, if more than one whale is initiating changes, there should be competing versions of novel songs. So far, there is no direct evidence of any vocally trendsetting humpbacks. Second, the progressive regularity of phrase changes across whales noted by Payne et al. (1983) is not actually explained by the song-copying hypothesis. Why would the vast majority of “innovations” to songs consist of subtle progressive shifts in one or more acoustic characteristics of a phrase? The changes that Payne and colleagues identified, according to them, “progressed in such a predictable fashion that far from looking like accidents of forgetfulness they appeared to follow set rules of progressive change.” Changes that follow prescribed rules are not creative changes. Why are the songs of some consecutive years “extremely similar” while songs from other consecutive years have few if any themes in common (Payne and Payne, 1985; Allen et al., 2018)? The song-copying hypothesis can only explain such cross-year fluctuations in the rate of change by adding auxiliary speculations, such as that innovators are more influential and wanderlusty in some years than others (Noad et al., 2000). Similarly, the song-copying hypothesis has little to say about why some themes (e.g., the surface ratchet theme) are more consistently recurrent than others, or more generally, why themes show any differences in the rate and direction of how they progressively change over time, either within or across songs. Such omissions become particularly problematic when groups of whales that are not in acoustic contact are simultaneously morphing their phrases in similar ways (Cerchio et al., 2001; Darling and Sousa-Lima, 2005; Mercado and Perazio, in press), and when the ways that they are morphing phrases match trajectories of phrase-change observed more than a decade earlier (Figures 2B–C). Finally, the possibility that a selective process of copying innovative variants of phrases would lead to the same phrases repeatedly emerging across populations and decades (Mercado and Perazio, in press) seems about as likely as that multiple species would go extinct only to suddenly reappear later on in the geological timeline.

**Figure 5:**
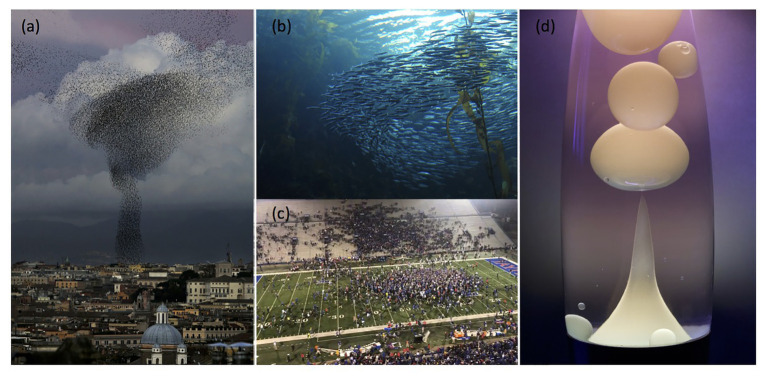
**(A)** Murmurating starlings and **(B)** schooling fish form morphing blobs that maintain dynamic cohesion when group members collectively react to the actions of their neighbors. **(C)** Fans may similarly coalesce in their movements when emotions ride high. **(D)** Even warmed wax can self-organize into complex, dynamic forms that progressively evolve over time based on relatively simple rules of thermodynamics.

Despite these limitations, the song-copying hypothesis has gone unchallenged for 40 years. And, the everchanging songs of humpback whales are often noted as one of the clearest and most impressive cases of cultural transmission (Rendell and Whitehead, 2001b; Laland and Janik, 2006). In part, this is because cetologists have “ruled out” alternative explanations for the dynamic changes evident within humpback whales’ songs that are linked to genetics, ecological conditions, or individual learning. Using that logic, however, Cerchio et al. (2001) ruled out song copying as a possible mechanism of song change by showing that singers converged in their morphing of phrases even in the absence of acoustic contact. In reality, none of these possibilities have been ruled out. Humpback whales may be born possessing “templates” that determine the kinds of phrases they can produce throughout their lives, as well as how they will morph phrases over time. Ecological factors, including the acoustic soundscape created by other singing whales, may modulate the rate at which singers morph phrases, even if singers are oblivious to the details of what other whales are singing. Singers may learn over time not only how to produce phrases more precisely, but also how to shift acoustic features in ways that increase the functionality or efficiency of songs, especially in contexts where multiple singers are audible. Recent evidence that humpbacks modify their singing in reaction to hearing the songs of other singers (Cholewiak et al., 2018), suggest that they can adjust their vocal actions dynamically based on the acoustic context. However, this does not imply that those adjustments involve any song copying or any evaluation of the quality of another whale’s song. In fact, many animals flexibly adjust their actions in response to those of conspecifics in ways that lead to complex, convergent action patterns, including schooling fish, murmurating birds, and swarming humans (Figure 5). Might such non-cultural mechanisms of social interaction account for how groups of humpback whales collectively change their vocal actions over time?

Like humpback whales, schools of fish and flocks of birds sometimes coordinate their actions over extended periods of time (Parrish et al., 2002; Hemelrijk and Hildenbrandt, 2011, 2012). For example, when birds murmurate, they may fly in blobs that slowly morph over time before suddenly shifting into more complex, yet still coherent configurations (Ballerini et al., 2008a,b; Hemelrijk and Hildenbrandt, 2011). Changes in the overall form of the flock are progressive and “evolve” in ways that are not strongly constrained by environmental conditions. There are no innovators, coordinators, or copiers in such flocks. Nevertheless, members of a flock progressively modify their collective movements in convergent ways. Local interactions can lead to the emergence of complex convergent patterns when those interactions are nonlinear (Ballerini et al., 2008a,b; Moussaid et al., 2009; Cavagna et al., 2010; Storms et al., 2019), often referred to as self-organization. In self-organizing systems, the rules that determine how individuals within a group act depend primarily on local information. Self-organization depends on positive and negative feedback loops, in which one change can lead to a chain reaction of additional changes. The emergence of novel patterns also depends on fluctuations in the actions of individuals within the group as well as multiple interactions between members of the group. Studies of collective actions in social insects have repeatedly shown that relatively simple actions of group members can lead to complex collective outcomes.

The dynamic movements of groups of birds and fish may seem ephemeral compared to the progressively changing songs of humpback whales, but self-organizing systems may also lead to cumulative changes in actions, especially when actions modify the group’s environment, a process known as stigmergy (Parrish et al., 2002; Moussaid et al., 2009). Stigmergic communication in insects often involves chemical or physical changes to the environment. In the case of humpback whales, stigmergic processes may lead to cumulative changes in the acoustic soundscapes experienced by singers. Such processes clearly depend on the social transmission of information, since the actions of others provide the stimuli that drive the adjustments in individual reactions. However, these reactions do not require vocal learning, an exchange of messages, evaluation of the relative goodness of others’ actions, or any memory of past actions. Within self-organizing systems relatively small perturbations at the individual level can lead to significant changes at the collective level (Moussaid et al., 2009). How such changes affect the behavior of the group can depend on many factors, including the density (and goals) of interacting individuals, their movements, and environmental conditions. Simplified interaction models can produce complex emergent patterns of everchanging convergence (Sole et al., 1999; Muñoz, 2018), showing that sophisticated cultural learning processes are not required to explain either progressive behavioral changes or convergence of changes within a group. That being said, given the known vocal imitation abilities of cetaceans (Mercado et al., 2014), and their impressive cognitive capacities (Mercado and Delong, 2010), it would be surprising if singing humpback whales are limited to genetically-determined reactions to the sound sequences they produce and experience. Consistent with this possibility, anecdotal observations suggest that singers can flexibly respond to unique sound sequences (Rothenberg, 2008). In this respect, the behavior of individual singers, especially in contexts where social interactions are limited, may be more comparable to the flexible, voluntary actions of humans than they are to the collective, reflexive reactions of fish or ants.

Notably, local interactive mechanisms provide a simple explanation for why the kinds of phrase morphing evident within shifting themes produced by singing humpback whales would be highly similar to the progressive changes that are observed across years, for why such changes might occur rapidly in some phrases and years but more slowly in others, and for why specific phrases would emerge, disappear, and re-emerge across years and populations. All of these phenomena are characteristic of nonlinear dynamic systems. Self-similarity at multiple scales is a typical feature of fractals in nature, as is scale invariance, where similar patterns are evident at different levels of analysis (Sole et al., 1999; Muñoz, 2018). Criticality in dynamic, self-organizing systems is associated with periods of stabilization and intermittent rapid changes in state (Sole et al., 1999; Muñoz, 2018), as is seen in the rapid, fluid changes in flocks of murmurating birds and fish schools interacting with a predator, followed by a gradual return to earlier configurations. Models that assume that selective copying of preferred innovations drives song transformations predict none of these dynamic patterns.

## Conclusion

Singing humpback whales are clearly changing their songs in complex ways over time. Relatively little is known about what determines how and when a singer will modify song features. It is also not known what prompts a singer to vary the duration of its songs within a song session, the number of times it repeats phrases within themes, or the phrases it morphs and to what extent. Faced with a litany of unanswered questions regarding why humpback whales sing the way they do, researchers have turned to cultural mechanisms as a potential answer to them all. While it is true that flexible social learning capacities can explain a wide range of complex social behaviors (Freeberg et al., 2012; Sewall, 2015), if cultural transmission becomes the default explanation for anything a singer does to change (or maintain) song characteristics, then it becomes a pseudoscientific explanation. Historically, the song-copying hypothesis has been attractive in part because it seems to tie the vocal ecology of humpback whales to that of other singing species like song-learning birds (Payne et al., 1983; Parsons et al., 2008; Herman, 2017; Cholewiak et al., 2018; Garland and McGregor, 2020). However, from the beginning, researchers have acknowledged that what whales are doing when they sing differs significantly in many ways from what singing birds are doing (Winn and Winn, 1978).

Recordings collected to date make it clear that singing humpbacks are not morphing their phrases arbitrarily. The ways in which they progressively morph phrases within and across songs does not match with what selectionist models of cultural evolution predict should happen, but neither do they match with a neutral model of evolutionary change in which random mutations spread through a population (Kimura, 1979). Evolutionary models of collective changes appear to be inadequate for characterizing the ways in which singers modify their songs over time (McLoughlin et al., 2018). Whether dynamic systems models of interacting agents can meet this challenge remains to be seen. Current models of self-organization focus heavily on variables that affect the movement patterns of individuals traveling within groups (Moussaid et al., 2009; Hemelrijk and Hildenbrandt, 2012), and less on communicative behavior (for exceptions, see Aihara et al., 2007; Ramírez-Avila et al., 2018). Nevertheless, approaches that have shed light on the variables that drive collective behavior in other animals can provide some clues as to the kinds of studies that might reveal similar local interaction mechanisms operating in singing humpback whales. For example, observational studies of detailed movements of individual birds within large murmurating flocks revealed that individuals adjusted their movements based on the actions of their six or seven closest neighbors (Hildenbrandt et al., 2010). Similar studies of birds’ responses to predators revealed that birds showed modal patterns of evasive maneuvers (Storms et al., 2019). Singing whales might similarly modulate their songs based on the actions of their nearest neighbors and might modulate their songs in predictable ways when nearby whales engage in specific acts (e.g., breaching, vocalizing within surface-active competitive pods, etc.). Other mysticetes are known to modulate their singing behavior in relation to their swimming speeds (Clark et al., 2019), and humpback whales may similarly sing differently depending on the actions they are engaged in while singing (Henderson et al., 2018). Long-term, multi-day monitoring of individual singers producing songs both alone and in various social contexts can potentially provide important new clues as to how individuals respond to the vocal and physical actions of other whales.

From the aural perspectives of human listeners, the characteristics of humpback whale songs are highly complex and enigmatically organized. These features have led to speculation about a variety of cognitive processes that singing humpbacks must possess to be able to produce such acoustic spectacles, including prodigious memory capacities (Guinee and Payne, 1988; Handel et al., 2012; Garland et al., 2017), creative proclivities (Payne, 2000), and imitative skills (Janik, 2009). They have also spawned numerous conjectures regarding why whales might have evolved such perplexing vocal acts (Payne et al., 1983; Parsons et al., 2008). Arguably, scientific efforts to clarify what singing humpback whales are doing, and why, have generated more heat than light given that Payne and McVay’s (1971) initial suggestion that perhaps female whales like fancy songs still tops the list of “explanations” for the phenomenon. Feminine fancies aside, there are likely proximate mechanisms that determine when a singer will produce longer or shorter song cycles (Chu and Harcourt, 1986; Miller et al., 2000; Fristrup et al., 2003), when they will skip or repeat themes (Frumhoff, 1983), and when they will morph phrase features, each of which can be experimentally and observationally investigated. For instance, if songs function collectively to act as a beacon for distant whales (Winn and Winn, 1978; Herman, 2017), then introducing multiple playbacks of current song around a targeted singer (at naturalistic distances) should have little effect on how the whale sings. Alternatively, if whales are adjusting their songs in response to the songs of their neighbors, then this intervention should have noticeable effects on the properties of the songs being produced (e.g., see Cholewiak et al., 2018). If novelty or peer pressure drive song changes (Garland et al., 2017), then having surrounding virtual whales all introduce a “new” theme (say from a distant population) should be sufficient to provoke at least some singers to adopt that theme. If instead singers adjust their songs to minimize cross-song interference (Mercado, 2018, 2020), then it should be possible to control how a singer responds to surrounding virtual singers by selecting the timing and spectral content of playbacks based on the songs that the singer is producing. Table 1 describes several key studies that could be done to clarify the role that either song copying or dynamic social interactions play in song transformations.

**Table 1:** Key predictions of the song-copying hypothesis and dynamic-interactions hypothesis along with potential approaches to testing those predictions.

Cultural transmission *via* song copying: “Singers copy high quality, innovative songs”	Song morphing through local interactions: “Singers modulate song features reactively”	How to test predictions
Song differences should grow with increasing temporal and/or geographical separation.	Themes should recur within and across populations.	These predictions can be tested through objective comparisons of recordings made across decades.
Controlled introduction of high quality, innovative songs will lead to copying.	Controlled introduction of foreign songs will not cause any singers to adopt those songs.	Testable through playbacks or by establishing direct communication channels between populations (e.g., two-way cellular transmission between acoustically isolated singers).
Song evolution will follow dissimilar trajectories across populations.	Song morphing will follow similar trajectories across populations.	Testable through objective comparisons of relative changes in unit and phrase characteristics across years in acoustically isolated populations.
In locales where multiple singers are audible, the most skilled singer will not change its song, while other group members may modify their songs to better match the highest quality song.	In locales where multiple singers are audible, all singers will modulate song production in predictable ways.	Can be tested by monitoring the songs of multiple singers or by artificially bringing lone singers into acoustic contact (e.g., using two-way cellular transmissions).
Singers should generally produce the highest quality song they are capable of producing, to consistently advertise their fitness.	Singers will continuously modulate song production based on the acoustic conditions or behavioral context within which they are singing.	Testable through objective analyses of intra-individual variations within and across song sessions.
Individual differences in songs should be most apparent when multiple singers are in direct acoustic competition.	Individual differences in songs should be more apparent when singers are alone and not constrained by the vocal actions of other singers.	Can be tested by objectively comparing songs produced by singers within choruses relative to songs produced by lone singers.

In the past, researchers have questioned whether any explanation other than cultural transmission can possibly account for the complex, collective changes observed in humpback whale songs. Payne et al. (1983, p. 85) noted that, “It is inconceivable that such rapid and complete turnover of the song material could reflect genetic changes.” Similarly, Rendell and Whitehead (2001a) argued that, “horizontal cultural transmission almost certainly plays an important role in maintaining song homogeneity as there is no conceivable environmental trigger for such a pattern of variation,” and, “the continuously evolving songs of humpback and bowhead whales have no conceivable environmental or genetic cause.” In the words of Inigo Montoya, in regard to things inconceivable, “You keep using that word. I do not think it means what you think it means.”

## Author Contributions

The author confirms being the sole contributor of this work and has approved it for publication.

### Conflict of Interest

The author declares that the research was conducted in the absence of any commercial or financial relationships that could be construed as a potential conflict of interest.

## Footnotes

^1^It is important to keep in mind that what counts as “the same” in whale songs differs from the criteria typically used in the bird song literature. For instance, a 5 min long song containing two themes (A and B) would be considered to be the same as a 30 min song containing four themes (A–D), as long as the two themes in the 5 min song contained phrases subjectively similar to those in two themes within the 30 min song. This is because the main criteria for two humpback whale songs to be considered “the same” is that the themes being produced in the song are part of the common set being used by whales in a population, occurring in the expected order. In fact, two 5 min songs that contained no sounds or patterns in common would be “the same” by these criteria, if say a third song contained only the C and D themes.
